# Single Center Experience With Pediatric Patients With GATA2 Deficiency

**DOI:** 10.3389/fped.2022.801810

**Published:** 2022-02-22

**Authors:** Galina Ovsyannikova, Anna Pavlova, Ekaterina Deordieva, Elena Raykina, Alexey Pshonkin, Alexey Maschan, Michael Maschan

**Affiliations:** ^1^Department of Pediatric Hematology and Oncology, Dmitry Rogachev National Medical Research Center of Pediatric Hematology, Oncology and Immunology, Moscow, Russia; ^2^Laboratory of Molecular Biology, Dmitry Rogachev National Medical Research Center of Pediatric Hematology, Oncology and Immunology, Moscow, Russia; ^3^Department of Pediatric Immunology, Dmitry Rogachev National Medical Research Center of Pediatric Hematology, Oncology and Immunology, Moscow, Russia; ^4^Department of Hematopoietic Stem Cell Transplantation, Dmitry Rogachev National Medical Research Center of Pediatric Hematology, Oncology and Immunology, Moscow, Russia

**Keywords:** myeloid neoplasms with germline predisposition, aplastic anemia, myelodysplastic syndrome, pediatric patients, GATA2 deficiency

## Abstract

GATA2 deficiency is one of the most common predisposing conditions for MDS in young individuals. It is characterized by autosomal dominant inheritance and a high rate of *de novo* mutations. Here we describe the clinical phenotype and hematological presentation of 10 pediatric patients with GATA2 deficiency presented to the Dmitry Rogachev Center between 2013 and 2020. All patients had been referred for neutropenia or suspected aplastic anemia. While some patients presented with an immunological phenotype, others displayed monosomy 7 and MDS. The clinical presentation with MDS in infancy and the constitutional phenotypes in our patients underline the great variability in clinical manifestation. Careful description of cohorts with GATA2 deficiency from different countries and genetic backgrounds will help to unravel the enormous heterogeneity of this recently discovered genetic disorder.

## Introduction

Establishing the correct diagnosis in children with cytopenia has recently become more challenging. Clinical and laboratory features of a number of genetic disorders, specifically germline disease predisposing to myeloid neoplasia, the classical inherited bone marrow failure syndromes (BMFS) and primary immunodeficiencies, can overlap. In addition, these inherited conditions need to be differentiated from acquired disorders such as aplastic anemia.

GATA2 deficiency is one of the most common predisposing conditions for MDS in young individuals. It is characterized by autosomal dominant inheritance with a high rate of *de novo* mutations. GATA2 syndrome exhibits incomplete penetrance and heterogeneity in clinical presentation with manifestation of myeloid neoplasia at any age (1). *GATA2* encodes a zinc finger transcription factor critical to early hematopoiesis, mononuclear development, and alveolar macrophage activity (2, 3). Heterozygous germline mutations in *GATA2* lead to complex and heterogeneous clinical phenotypes including MonoMAC syndrome (monocytopenia and mycobacterial infections)/DCML deficiency (dendritic cell, monocyte, B and natural killer (NK) lymphoid deficiency) (2–7), lymphedema (Emberger syndrome) (8, 9) and familial MDS/AML (10). In addition, other recurrent phenotypes have been described such as primary pediatric MDS (11), chronic neutropenia (12), aplastic anemia (13), pulmonary alveolar proteinosis (14), dermatological (15), autoimmune or vascular features (16).

A continuous review of clinical and genetic data on patients with GATA2 deficiency in different parts of the world will help to understand the heterogeneity of presenting features. Careful description of patient series with this rare disorder will facilitate the development of standards of care for patients and healthy gene carriers. This manuscript describes the clinical and hematological phenotype of 10 children with GATA2 deficiency diagnosed at the Dmitry Rogachev National Research Center in Moscow.

## Materials and Methods

Peripheral blood samples from patients or family members had been obtained with informed consent. The study was approved by the local ethics committee. Metaphase karyotyping, chromosome banding analyses of diagnostic bone marrow specimens were performed according to standard procedures. Genetic testing was done by Sanger sequencing for single gene analysis or target next-generation sequencing on the MiSeq/NextSeq (Illumina, USA) using BMFS custom gene panel. The BMFS custom gene panel consists of 197 genes (Supplementary Table 1). Population frequency evaluation of the identified variants was performed using the data from the gnomAD Exomes and gnomAD Genomes projects. Computational pathogenicity assessment of missense variants was performed using the following prediction tools: SIFT, Provean, PolyPhen-2, and UMD Predictor. Computational effect prediction of the splice site and splice site region alterations was performed with Human Splicing Finder 3.0 and NNSplice. Evaluation of variant clinical relevance was performed using Online Mendelian Inheritance in Man and the Human Gene Mutation Database. The clinical significance of the identified variants has been assigned based on the American College of Medical Genetics and Genomics recommendations (17).

Characteristics of hematopoietic stem cell transplantation are presented in the Supplementary Table 2.

## Results

GATA2 deficiency was diagnosed in 10 patients referred for a diagnosis of cytopenia between 2013 and 2020 (Table 1). In all 10 patients *GATA2* mutations located in zinc finger 2; mutations were missense in 4 cases, and truncating in 6 with 4 nonsense and 2 frameshift changes. Genetic analysis in non-hematological tissue demonstrated germline origin in all six cases analyzed.

**Table 1:** Clinical and hematological characteristics of patients with GATA2 deficiency.

**Pt #**	**Sex**	**GATA2 mutation**	Age^*^yrs	CBC^*^					**BM** ^**^			Karyo-type^**^	**Constitutional phenotype**	**Infections**	**Progression to MDS-EB, AML**	**Family history**	**HSCT**	**At last follow-up**	
				**WBC G/L**	**ANC G/L**	**Hb g/L**	**MCV fL**	**Plt G/L**	**Cell Content**	**Blast %**	**Dysplasia**							**Age, yrs**	**Status**
1	F	Ex 6 c.1187G>A p.R396Q	14.9	2.5	0.5	10.1	107	319	hypo	<5%	E, G	N	–	+	–	Mother: breast cancer, dead^%^	–	17.1	Alive
2	F	Ex 5 c.1033_1060del p.A345Rfs^*^33	10.1	2.4	1.1	11.3	94	141	hypo	<5%	E,G	N	+	+	–	None	–	14.9	Dead
3	M	Ex 5 c.1082G>A p.R361H	17.6	1.6	0.32	8.9	101	469	hypo	<5%	E	+8	+	+	–	None^§^	+	19.8	Alive
4	F	Ex 5 c.1082G>A p.R361H	2.8	10.2	0.52	11.8	83.8	106	hypo	<5%	E,G,M	−7	+	+	–	None^§^	+	4.5	Alive
5	M	Ex 5 c.1084C>T p.R362^*^	10.0	2.5	0.5	12.3	–	319	hypo	<5%	E	−7	+	+	–	Father: lymphedema, stomach cancer, dead (30 yrs)^%^	+	17.3	Alive
6	M	Ex 5 c.1084C>T p.R362^*^	8.7	1.9	0.6	9.4	–	169	hypo	<5%	E, G	−7	_+_	+	–	None^§^	–	9.7	Alive
7	F	Ex 5 c.1084C>T p.R362^*^	10.7	1.6	0.7	11.0	88.5	134	normo	<5%	E	N	+	+	–	None^§^	–	20.9	Alive
8	M	Ex5, c.1084C>T p.R362^*^	7.8	1.6	0.8	7.2	88.5	143	normo	<5%	ND	−7	–	+	+	None	–	9.2	Dead
9	M	Ex6, c.1187G>A p.R396Q	10.0	1.2	0.5	11.2	97	152	hypo	<5%	G	N^&^	+	+	–	None^§^	+	15.9	Dead
10	M	Int 5, c.1144-2A>C p.?	12.5	2.25	0.4	14.6	86.2	215	hypo	<5%	G	ND	+	+	–	None^§^	–	13.5	Alive

*N, normal; ND, no data; BM, bone marrow; M, Male; F, Female; CBC, complete blood count; WBC, white blood cells; ANC, absolute neutrophil count; Plt, Platelets. E, erythropoiesis; G, granulopoiesis; M, megakaryopoiesis*;

*, *at onset of cytopenia*;

**, *karyotype at first bone marrow analysis performed*;

&, *patient developed −7 and +8; §, GATA2 wildtype in parents*;

*^%^,GATA2 mutational study not performed*.

Eight of the 10 patients had physical abnormalities (Table 2); 5 of them showed an Emberger-like phenotype with deafness and/or lymphedema/hydrocele, 3 had abnormalities of the genitourinary tract, 2 constitutional heart disease and one patient gallbladder anomaly. Genetic evaluation of family members established the *de novo* origin of the identified mutation in 6 cases (Table 1). The father of one patient was known to have lymphedema, but mutational analysis was not feasible due to absent material.

**Table 2:** Constitutional phenotype and infections.

**Pt #**	**Sex**	**GATA2 mutation**	**Constitutional phenotype**	**Infections**
1	F	c.1187G>A p.R396Q	–	Chronic pyelonephritis, recurrent bronchitis
2	F	c.1033_1060del p.A345Rfs^*^33	Deafness; biliary tract anomaly with gallbladder agenesis and hepatomegaly; 5 café au lait spots	Recurrent otitis and bronchitis, pneumonia, cellulitis, furunculosis, suppurative lymphadenitis, fatal varicella encephalitis
3	M	c.1082G>A p.R361H	Right kidney hypoplasia; congenital hypospadia	Chronic pyelonephritis
4	F	c.1082G>A p.R361H	Lymphedema of right foot; hydronephrosis of the left kidney with left sited vesicoureteral reflux and megaureter	Chronic pyelonephritis
5	M	c.1084C>T p.R362^*^	Lymphedema of lower limbs	Recurrent tonsillitis and bronchitis, pneumonia
6	M	c.1084C>T p.R362^*^	Minor cardiac abnormalities not further specified, talipes valgus	Sinusitis, otitis, adenoiditis.
7	F	c.1084C>T p.R362^*^	Ventricular septal defect, pulmonary valve stenosis; idiopathic hypothyroidism	Recurrent bronchitis and pneumonia, cellulitis, CMV-, EBV-viremia.
8	M	Ex5, c.1084C>T p.R362^*^	–	Recurrent bronchitis and pneumonia
9	M	c.1187G>A p.R396Q	Hypoplasia of the right kidney, hydrocele and spermatocele on the left side	Recurrent bronchitis, pneumonia, furunculosis, warts
10	M	c.1144-2A>C p.?	Syndactyly type I-b Deafness	Recurrent bronchitis and otitis, pneumonia,

*M, Male; F, Female*;

*, *termination codon*.

Median age at onset of cytopenia was 10.5 years (range 2.8–17.6), and the median duration between first cytopenia and consultation in our referral center was 2.8 years (range 0.1–10.2). Leukopenia (9/10 patients), neutropenia (10/10) and profound monocytopenia (10/10) were the most prevalent hematological manifestations; median values for the absolute neutrophil and monocyte count were 0.59 G/L (range 0.32–1.10) and 0.06 G/L (range 0.01–0.18), respectively (Table 1). In contrast, none of the patients had a platelet count below 100 G/L (range 106–469, median 217). Median hemoglobin level was 10.8 g/dl (range 7.2–14.6), one patient required red cell transfusions prior to diagnosis. Red cells were macrocytic for age in 8/10 patients. In all patients, the bone marrow blast percentage was <5%, and in 8 out of the 10 cases the marrow was hypocellular. Chromosomal analysis showed monosomy 7 in 4/10 patients, one patient had trisomy 8 (Table 1).

Recurrent or severe infections at diagnosis or during the clinical course were observed in all patients. One patient (Pt 2) succumbed to varicella zoster encephalitis 4.9 years after diagnosis; she had a normal karyotype, normal IgG levels, but a profound decrease in B-cells as well as neutropenia and monocoytopenia. In 6 patients immunoglobulin G serum levels were available and within the normal range. The analysis of lymphocyte subpopulations showed a reduction in number of B-cells (CD19+; median 0.02 G/L, range 0.01–0.03) and NK-cells (CD16+/CD56+; median 0.052 G/L, range 0.013–0.124) in the five patients studied; 3 of these patients also demonstrated T-cell deficiency (CD3+, median 1.14 G/L, range 0.44–1.71).

Allogeneic hematopoietic stem cell transplantation (HSCT) was not readily available for all patients. Four of the 10 patients were transplanted a median time of 4.0 years (range 0.5–6.7) from onset of cytopenia (Supplemental Table 2). Two of these 4 patients had presented with monosomy 7, one with trisomy 8, and none of them had an increased blast percentage. One patient was grafted from a matched unrelated donor (MUD), while the other 3 patients received a haploidentical graft. In 2 of the 3 patients with a haploidentical parental transplant, a second grafting procedure was necessary because of primary graft failure.

Six patients were followed in the absence of HSCT. Median time from onset of cytopenia to last follow-up was 3.9 years (range 1.0–10.2). One of these patients (Pt 8) experienced disease progression with increase in blast percentage 1.2 years from onset of cytopenia. He was found to have monosomy 7, progressed rapidly to AML and succumbed to candida sepsis shortly thereafter. In one patient (Pt 9), monosomy 7 with trisomy 8 was diagnosed 5.7 years after hematological presentation.

At the time of writing seven patients are alive, one patient died due to varicella zoster encephalitis (Pt 2), one patient died after the second HSCT due to infectious complications (Pt 9), and another one due to infectious complications during intensive chemotherapy (Pt 8) (Table 1).

## Discussion

All patients with GATA2 deficiency presented in this retrospective cohort had neutropenia. More strikingly, the absolute neutrophil count in all patients was severely decreased with a range between 0.32–1.10 G/L at time of first evaluation. In contrast to what has been reported in the largest series of GATA2 deficiency in pediatric MDS (1–11), all patients presented here had monocytopenia with an absolute monocyte count ranging between 0.01 and 0.18 G/L. Also, thrombocytopenia and anemia were less common, likely indicating that some patients presented here had an immunodeficiency - type of manifestation rather than an MDS-type presentation.

Median age at diagnosis in the largest series of GATA2 deficiency in pediatric MDS was 12 years, in fact, all patients were above the age of 3 years at presentation (11). In this consecutive study of EWOG-MDS patients, 70% of GATA2 deficient cases had monosomy 7. Infants with MDS and monosomy 7 are more likely to have an underlying SAMD9/SAMD9 syndrome than GATA2 deficiency (18). The youngest patient included in the series presented here, was 2.8 years and had monosomy 7 at diagnosis. This observation emphasizes that despite the different age distribution in presentation between GATA2 deficiency and SAMD9/SAMD9L syndrome, all patients need complete diagnostic work-up for all genetic disorders irrespective of age.

Physical examination with constitutional abnormalities in 8 of the 10 patients had already raised the suspicion of a genetic disorder prior to molecular testing. Interestingly, 3 of the 8 patients had abnormalities of the genitourinary tract, possibly indicating that this organ system is more commonly affected than previously appreciated (8–16). In addition, we describe biliary tract and cardiac anomalies as potential novel constitutional features of GATA2 deficiency. The exact description of the spectrum of constitutional abnormalities related to GATA2 deficiency will require large carefully selected patient cohorts. For instance, although café au lait spots were noted in one of our patients and had previously been described in GATA2 deficiency (19), they are also not very common in the general population. Furthermore, syndactyly of the foot in one of our patients is likely not related to GATA2 deficiency since his father with GATA2 wild type had syndactyly of his fingers.

Taking into account that more than 80% of patients with GATA2 deficiency present with a hematologic malignancy around the age of 40 years (16), HSCT is generally considered early in the clinical course. Indeed, most patients with MDS and monosomy 7 are transplanted as soon as a suitable donor is available. Progression to MDS and AML may be very rapid as observed in one of our patients who died from infectious complications while receiving intensive chemotherapy for AML. Also, profound neutropenia or other signs of immunodeficiency should result in timely HSCT to avoid severe infectious complication. One of our patients died from severe varicella infection. If a MUD is not available, a haploidentical HSCT can be performed. Stem cell source and type of donor does not significantly influence HSCT outcome in GATA2 deficiency, while abnormal karyotype and blast percentage are the main risk factors for poor outcome (20). HSCT must be carefully considered in all individuals with GATA2 deficiency, and timely grafting when indicated will most likely improve outcome for this group of patients.

## Conclusions

In this retrospective study, we expand the clinical and genetic phenotype of GATA deficiency by outlining previously undescribed constitutional abnormalities and novel mutations. Thus, our work underlines the broad heterogeneity of the predisposition syndrome.

## Data Availability Statement

The NGS datasets presented in this study are available upon request from the author (Galina Ovsyannikova) by email: Galina.Ovsyannikova@fccho-moscow.ru.

## Ethics Statement

The studies involving human participants were reviewed and approved by the Local Ethics Committee of Dmitry Rogachev National Research Center of pediatric oncology, hematology and immunology. Written informed consent to participate in this study was provided by the participants' legal guardian/next of kin.

## Author Contributions

GO collected and analyzed data and wrote the manuscript. AP and ER performed genetic data analysis. ED and AP participated in data collection. AM and MM reviewed the manuscript and supervised the project. All authors contributed to the study design.

## Funding

This study is supported by a grant from the Charitable Foundation Science for Children in the Russian Federation.

## Conflict of Interest

The authors declare that the research was conducted in the absence of any commercial or financial relationships that could be construed as a potential conflict of interest.

## Publisher's Note

All claims expressed in this article are solely those of the authors and do not necessarily represent those of their affiliated organizations, or those of the publisher, the editors and the reviewers. Any product that may be evaluated in this article, or claim that may be made by its manufacturer, is not guaranteed or endorsed by the publisher.
