# Supplementary material for: Single Center Experience With Pediatric Patients With GATA2 Deficiency
Source: Front Pediatr. 2022 Feb 22;10:801810. doi: 10.3389/fped.2022.801810 (PMC8901576; doi:10.3389/fped.2022.801810)
Supplement: Supplementary file 1 [file Table_1.DOCX]

Supplementary Material

**Supplementary table 1S.** The Bone Marrow Failure Syndrome custom gene panel.

| **The Bone Marrow Failure Syndrome custom gene panel consists of 197 genes** |
| --- |
| ABCD4, ACD, AK2, ALAS2, ANKRD26, AP3B1, ASXL1, ATM, ATR, ATRX, BCOR, BCORL1, BLM, BRAF, BRCA1, BRCA2, BRIP1, C15ORF41, CA2, CALR, CBL, CBLB, CBLIF, CDAN1, CDKN2A, CEBPA, CLCN7, COG6, COQ2, CSF3R, CTC1, CXCR4, DDX41, DKC1, DNAJC21, DNMT3A, EFL1, ELANE, ERCC4, ERCC6L2, ETV6, EXOC3L2, EZH2, FANCA, FANCB, FANCC, FANCD2, FANCE, FANCF, FANCG, FANCI, FANCL, FANCM, FAS, FLT3, G6PC3, GAR1, GATA1, GATA2, GFI1, GLA, GNAS, HAX1, HOXA11, HRAS, IDH1, IDH2, IFNGR2, IKZF1, IVD, JAGN1, JAK2, JAK3, KDM6A, KIT, KLF1, KRAS, LIG4, LMBRD1, LRP5, LYST, LZTR1, MAGT1, MAP2K1, MAP2K2, MARS, MECOM, MLH1, MMAA, MMAB, MMACHC, MMADHC, MMUT, MPIG6B, MPL, MTRR, MYD88, MYH9, MYSM1, NAF1, NBN, NF1, NHP2, NOP10, NPM1, NRAS, OSTM1, PALB2, PARN, PCCA, PCCB, PDGFRA, PLEKHM1, POT1, PRDX1, PRF1, PTEN, PTPN11, RAB27A, RAD21, RAD51, RAD51C, RAF1, RASA2, RBM8A, RIT1, RMRP, RPL10A, RPL11, RPL15, RPL18, RPL19, RPL26, RPL27, RPL3, RPL31, RPL34, RPL35, RPL35A, RPL36, RPL4, RPL5, RPL9, RPLP0, RPS10, RPS15, RPS15A, RPS17, RPS19, RPS24, RPS26, RPS27, RPS27A, RPS28, RPS29, RPS7, RRAS, RTEL1, RUNX1, SAMD9, SAMD9L, SBDS, SBF2, SEC23B, SETBP1, SF3B1, SH2B3, SH2D1A, SLC46A1, SLX4, SMARCAL1, SNX10, SOS1, SRP72, SRSF2, STAG2, TCIRG1, TERC, TERT, TET2, TFR2, TGFB1, TINF2, TNFRSF11A, TP53, TSR2, U2AF1, UBE2T, USB1, VPS13B, VPS33A, VPS45, WAS, WRAP53, WT1, XRCC2, ZRSR2. |
