# Supplementary material for: Single Center Experience With Pediatric Patients With GATA2 Deficiency
Source: Front Pediatr. 2022 Feb 22;10:801810. doi: 10.3389/fped.2022.801810 (PMC8901576; doi:10.3389/fped.2022.801810)
Supplement: Supplementary file 2 [file Table_2.DOCX]

Supplementary Material

**Supplementary table S2.** Characteristics of Hematopoietic Stem Cell Transplantation

| **Pt**  **#** | **Sex** | **Age (years)**  **at HSCT** | **Time from onset of cytopenia-**  **HSCT** | **Type of donor** | **Conditioning regimen** | **Primary**  **Engraftment (days)** | **Second HSCT** | **Type of donor** | **GVHD**  **acute grade**  **II-IV (1. or 2. HSCT)** | **At last follow-up**  **from 1. (2.) HSCT** | |
| --- | --- | --- | --- | --- | --- | --- | --- | --- | --- | --- | --- |
|  |  |  |  |  |  |  |  |  |  | **Days** | **Status** |
| 3 | M | 18.1 | 0.5 | MUD (10/10) | Bu, Cy, Mel | yes (21) | - | - | no | +790 | Alive |
| 4 | F | 4.2 | 1.4 | MMRD | Treo, Flu, Mel | yes (18) | - | - | II | +425 | Alive |
| 5 | M | 16.7 | 6.7 | MMRD | Treo, Flu, Thio | no | Yes | UD (10/10) | II | + 201  (+68) | Alive |
| 9 | M | 15.6 | 5.6 | MMRD | Treo, Flu, Thio | no | Yes | MMRD | no | + 79  (+31) | Dead |

MSD, matched sibling donor; MUD, matched unrelated donor; MMRD, mismatched (haploidentical) related donor; GVHD, graft-versus-host disease;

HSCT was performed in 4 out of 10 patients. The source of stem cells was bone marrow from a matched sibling donor (MUD; n = 1) and peripheral blood from a haploidentical related donor (MMRD; n = 3). Patients 5 and 9 received the second HSCT from a MUD (n=1) and a MMRD (n=1), respectively.

Conditioning regimen for the first transplant for patient 3 included busulfan, cyclophosphamide and melphalan.

For patient 4 it was treosulfan (42 g/m^2^ total dose; days -6, -5 and -4), fludarabine (150 mg/m^2^ total dose; days -6, -5, -4, -3, and -2) and melphalan (140 mg/m^2^ total dose; days -1). Graft-versus-host disease (GVHD) prophylaxis included cyclophosphamide (100 mg/kg total dose; days +3, +4), cyclosporine (from day +5), tocilizumab (day -1) and abatacept (day -1).

For patients 5 and 9 conditioning regimen consisted of treosulfan (42 g/m^2^ total dose; days -5, -4 and -3), thiotepa and (300 mg/m^2^ total dose; day - 2) and fludarabine (150 mg/m^2^ total dose; days -6, -5, -4, -3, and -2). In addition patients received serotherapy with rabbit antithymocyte globulin (ATG;) (5 mg/kg; days -5 and -4).

On day - 1 all patients received 375 mg/m^2^ rituximab for reducing the risk of Epstein-Barr virus related post-transplant lymphoproliferative disease.

Grafts from a MMRD (Pts 5 and 9) were TCRab^+^/CD19^+^ depleted by using an immunomagnetic method in accordance with the manufacturer’s instructions (Miltenyi Biotec, Bergisch Gladbach, Germany).
